# Supplementary material for: Identification of photoperiod-induced specific miRNAs in the adrenal glands of Sunite sheep (Ovis aries)
Source: Front Vet Sci. 2022 Jul 22;9:888207. doi: 10.3389/fvets.2022.888207 (PMC9354845; doi:10.3389/fvets.2022.888207)
Supplement: Supplementary file 1 [file Data_Sheet_1.ZIP › Supplementary file/miRDeep2 file.zip!/miRDeep2 file/Know_Structure/LP42A3/oar-mir-99a.pdf]

miRBase precursor	: oar-mir-99a
Total read count	: 605610
oar-miR-99a read count	: 604815
remaining reads	: 795

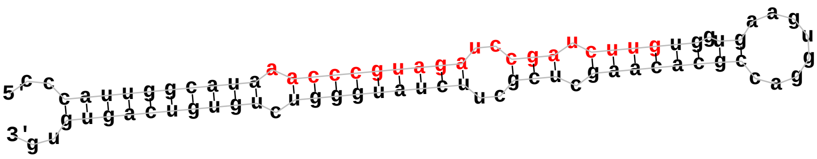

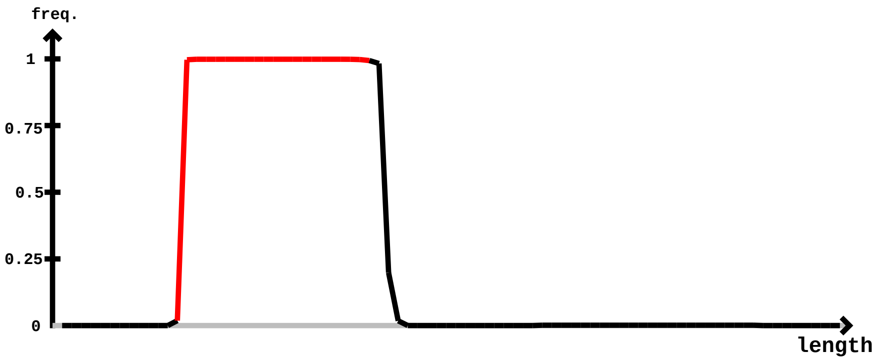

oar-miR-99a

5'- cccauuggcauaaacccguagauccgaucuuguggugaaguggaccgcacaagcucgcuucuaugggucugugucagugug -3' exp

..((((((((((.(((((((((..(((.((((((.((........)))))))).)))..))))))))).))))))))))..	reads	mm	sample
..........uaaacccguagauccgaucuugu..	2	0	seq
...........aaacccguagauccgauc..	2	0	seq
...........aaacccguagauccgaucu...	9	0	seq
...........aaacccguagauccgaucuu..	40	0	seq
...........aaacccguagauccgaucuug...	126	0	seq
...........aaacccguagauccgaucuugu..	10794	0	seq
...........aaacccguagauccgaAcuugu..	6	1	seq
...........aaacccguagauccgaAcuugug...	1	1	seq
...........aaacccguagauccgaucuugug...	268	0	seq
...........aaacccguagauccgaucuugugg..	18	0	seq
............aacccguagauccgauc..	108	0	seq
............aacccguagauccgaAc..	2	1	seq
............aacccguagauccgaucu...	577	0	seq
............aacccguagauccgaAcu...	43	1	seq
............aacccguagauccgaucuu..	2158	0	seq
............aacccguagauccgaAcuu..	102	1	seq
............aacccguagauccgaucuug...	5960	0	seq
............aacccguagauccgaAcuug...	571	1	seq
............aacccguagauccgaAcuugu..	13337	1	seq
............aacccguagauccgaucuugu..	450251	0	seq
............aacccguagauccgaucuugug...	86838	0	seq
............aacccguagauccgaAcuugug...	21584	1	seq
............aacccguagauccgaucuugugg..	10463	0	seq
............aacccguagauccgaAcuugugg..	288	1	seq
............aacccguagauccgaAcuuguggu...	1	1	seq
............aacccguagauccgaucuuguggu...	36	0	seq
.............acccguagauccgaAcu...	1	1	seq
.............acccguagauccgaucuu..	2	0	seq
.............acccguagauccgaAcuug...	2	1	seq
.............acccguagauccgaucuug...	8	0	seq
.............acccguagauccgaAcuugu..	133	1	seq
.............acccguagauccgaucuugu..	480	0	seq
.............acccguagauccgaAcuugug...	399	1	seq
.............acccguagauccgaucuugug...	106	0	seq
.............acccguagauccgaAcuugugg..	10	1	seq

oar-miR-99a

cccauuggcauaaacccguagauccgaucuuguggugaagguggaccgcacaagcucgcuucuaugggucugugucagugug			
.............acccguagauccgaucuugugg..	17	0	seq
..............cccguagauccgaucuugu..	12	0	seq
..............cccguagauccgaAcuugug...	1	1	seq
..............cccguagauccgaucuugug...	1	0	seq
...............ccguagauccgaucuug...	1	0	seq
...............ccguagauccgaAcuugu..	1	1	seq
...............ccguagauccgaucuugu..	21	0	seq
...............ccguagauccgaAcuugug...	2	1	seq
...............ccguagauccgaucuugug...	5	0	seq
...............ccguagauccgaucuugugg..	2	0	seq
................cguagauccgaucuugu..	22	0	seq
................cguagauccgaucuugug...	4	0	seq
....acaagcucgcuucuaugggucu...........	1	0	seq
....caagcucgcuucuaugggu.............	1	0	seq
....caagcucgcuucuaugggucu...........	5	0	seq
....caagcucgcuucuaugggucug..........	16	0	seq
....caagcucgcuucuaugggucugu.........	739	0	seq
....caagcucgcuucuaugggucugug........	3	0	seq
....caagcucgcuucuaugggucugugu.......	1	0	seq
....aagcucgcuucuaugggucu...........	1	0	seq
....aagcucgcuucuaugggucug..........	1	0	seq
....aagcucgcuucuaugggucugu.........	26	0	seq
....aagcucgcuucuaugggucugug........	1	0	seq
